# Neovascular age-related macular degeneration without exudative recurrence over 24 months after initial remission

**DOI:** 10.1038/s41598-022-19400-4

**Published:** 2022-09-19

**Authors:** Han Joo Cho, Young Joon Jeon, Wontae Yoon, Jihyun Yoon, Jaemin Kim, Jong Woo Kim

**Affiliations:** Kim’s Eye Hospital, Konyang University College of Medicine, 156, 4ga, Yeongdeungpo-dong, Yeongdeungpo-gu, Seoul, South Korea

**Keywords:** Outcomes research, Macular degeneration

## Abstract

We investigated the characteristics of neovascular age-related macular degeneration (AMD), which rarely recurs after initial remission. This study retrospectively analyzed 392 neovascular AMD patients treated with anti-vascular endothelial growth factor (VEGF). All patients received three monthly loading doses of anti-VEGF injections, followed by a pro re nata (as needed) regimen for 24 months. The baseline characteristics associated with the odds of having no recurrence within 24 months were evaluated using multivariate modeling. After the initial three loading injections over 24 months, 58 (14.8%) eyes showed no exudative recurrence and did not require additional anti-VEGF injections. These patients without exudative recurrence had significantly better best-corrected visual acuity (*P* = 0.003) and lower central subfoveal thickness (*P* = 0.035) at 24 months than those with exudative recurrence. Additionally, the incidence of macular atrophy was significantly lower in the former than in the latter (8.6% vs. 21.9%; *P* = 0.020). Multivariate analysis revealed that younger age (odds ratio [OR], 0.901; *P* = 0.033), smaller lesion size (OR, 0.589; *P* = 0.016), and absence of fibrovascular pigment epithelial detachment (PED) (OR, 1.349; *P* = 0.028) were associated with higher odds of no recurrence during follow-up. Approximately 15% of the neovascular AMD patients showed no exudative recurrence after initial remission during the 24-month follow-up. The infrequent recurrence after initial remission correlated with younger age, smaller lesion size, and absence of fibrovascular PED.

## Introduction

Age-related macular degeneration (AMD) is the leading cause of blindness among elderly people in developed countries^1^. Neovascular AMD is an advanced form of AMD characterized by the development of macular neovascularization (MNV)^2^. Since vascular endothelial growth factor (VEGF) is a key factor promoting neovascularization^3^, intravitreal anti-VEGF injections have been the main treatment option for neovascular AMD for over a decade^4^.

Anti-VEGF therapy comprises several treatment regimens for the management of neovascular AMD. Monthly fixed injection regimens are mainly used in clinical trials^5^. In real-world practice, the pro re nata (PRN; as-needed injection) and treat-and-extend (TAE) regimens are commonly used to reduce the treatment burden on patients. Patients receiving the TAE regimen are treated for extended intervals in case of dry macula, and the treatment interval is shortened when exudation is detected. The maximal interval between injections in the TAE protocol is generally 12 weeks^6,7^. Although TAE involves fewer injections, it is reportedly as effective as a monthly fixed injection regimen administered for 24 months. Therefore, the TAE regimen is currently the primary treatment strategy for patients with neovascular AMD^8^. When treating neovascular AMD with intravitreal anti-VEGF injections, the requirement for additional treatment varies among patients regardless of the treatment protocols^9–11^.

The Comparison of Age-related Macular Degeneration Treatment Trials (CATT) evaluated a subset of neovascular AMD patients with prolonged remission without additional anti-VEGF treatment. The study reported that 14.8% (*n* = 96) of the patients did not require additional treatment after the end of the 2-year treatment up to the 5-year follow-up completion. Additionally, among these 96 patients, 43 were treated with the PRN protocol^9^. For such neovascular AMD patients who maintain long-term remission without the need for additional injection, the TAE or fixed regimen could be an excessive or increased treatment burden.

To date, the characteristics of neovascular AMD cases without recurrence in the long term after initial loading injections have not been reported sufficiently. The prediction of infrequent recurrence may help provide a tailored approach to administer anti-VEGF treatment in patients with neovascular AMD. This study aimed to analyze the characteristics of a subset of neovascular AMD that does not recur after initial remission.

## Materials and methods

The AMD database of Kim’s Eye Hospital was reviewed through a computerized search. Patients who were diagnosed with neovascular AMD and had received intravitreal anti-VEGF therapy between January 2016 and March 2019 were identified retrospectively. The study was conducted in accordance with the principles of the Declaration of Helsinki and was approved by the Institutional Review Board of Kim’s Eye Hospital for the review and analysis of patient data. The requirement for informed consent from the patients was waived by the Institutional Review Board.

### Study population

The inclusion criteria for this study were as follows: (1) age > 50 years; (2) active MNV confirmed by multimodal imaging including spectral-domain optical coherence tomography (SD-OCT), fluorescein angiography (FA), and indocyanine green angiography (ICGA) at the first visit; (3) treatment-naïve MNV treated with anti-VEGF with ranibizumab (0.5 mg/0.05 mL) or aflibercept (2 mg/0.05 mL) using the PRN regimen; and (4) 24-month follow-up completed after the initial diagnosis.

The exclusion criteria were as follows: (1) treatment with a fixed regimen or proactive treatment, such as TAE; (2) foveal scars or fibrosis at the initial evaluation; and (3) concomitant retinal disorders that could affect visual outcomes, including high myopia, diabetic retinopathy, or retinal vein occlusion. When a patient had bilateral neovascular AMD, only the eye with the earlier diagnosis was included in the analysis.

All patients with neovascular AMD had received consecutive monthly loading anti-VEGF injections. After the initial loading injections, the patients were followed up for 2 years at intervals of 4–8 weeks. Based on the SD-OCT findings, additional PRN regimen anti-VEGF injections were administered when exudative recurrence was detected. Standardized examinations, including best-corrected visual acuity (BCVA), intraocular pressure, fundus examination, and SD-OCT (comprising 31 horizontal lines in 6 mm × 6 mm area) were performed at every visit for all the patients.

### Image analysis

Two retinal specialists (W.Y. and J.K.) independently analyzed all SD-OCT scans of the enrolled patients to detect exudative recurrence during the 24-month maintenance phase. Subretinal fluid (SRF) was defined by hyporeflective spaces between the photoreceptor layer and retinal pigment epithelium (RPE) on the SD-OCT images, while intraretinal fluid (IRF) was defined by hyporeflective spaces within the neurosensory retina^12^. Other macular changes, including subretinal hyperreflective material (SHRM)^13^ and retinal hemorrhage, were also evaluated.

Initial remission after anti-VEGF treatment was defined as complete resolution of the macular fluid, including SRF and IRF, after the three loading injections. Persistent pigment epithelial detachment (PED) was not considered a criterion indicating initial remission. Exudative recurrence was defined as the occurrence of SRF, IRF, retinal hemorrhage, or SHRM on SD-OCT after initial remission.

Various baseline characteristics of the neovascular AMD patients were analyzed. Using SD-OCT and FA/ICGA images, the lesions were classified as type 1 MNV (sub-RPE choroidal neovascularization [CNV]), type 2 MNV (subretinal CNV), or type 3 MNV (retinal angiomatous proliferation [RAP])^2^. Type 1 MNV cases with typical polypoidal lesions on ICGA were further classified as polypoidal choroidal vasculopathy (PCV; aneurysmal type 1 MNV)^2^. Additionally, the lesions were localized, and the lesion size was manually measured on the FA/ICGA image. Central foveal thickness was measured as the distance between the internal limiting membrane and surface of the RPE at the foveal center, while subfoveal choroidal thickness was measured as the distance from the outer portion of the hyperreflective line of the RPE to the hyporeflective line of the sclerochoroidal interface at the foveal center. All measurements were estimated using built-in software of the HRA-2 machine (Heidelberg Eye Explorer software, version 6.0.9.0; Heidelberg Engineering).

The PED definition used in this study was in line with that of our previous investigations and other clinical trials^14–16^, i.e., RPE elevation > 400 µm in width and > 75 µm in height or RPE elevation > 200 µm in vertical height. Furthermore, based on the SD-OCT findings, PED was classified as fibrovascular, when there was moderately reflective space adherent under the surface of the PED, or serous, when the PED was optically clear^15,17^. When the PED subtypes could not be determined on SD-OCT, the dominant component of the PED was evaluated using the corresponding FA/ICGA images.

### Visual and anatomical outcomes

The change in BCVA (converted from Snellen visual acuity to the logarithm of the minimal angle of resolution for statistical analysis) from baseline to 3, 6, 12, 18, and 24 months post-treatment was determined as the visual outcome. The proportion of patients who gained or lost more than three lines of BCVA when compared with the baseline was evaluated. Additionally, those with BCVA > 20/40 or < 20/200 after the treatment were evaluated.

The change in the central foveal thickness and incidence of macular atrophy (MA) during the study period were assessed as the anatomical outcomes. Based on the method reported in our previous publications, the development of MA was evaluated at 24 months from baseline^18,19^. In case of a hypopigmented area > 250 µm within the macular vascular arcades, the lesion was determined as MA after confirming the following: (1) visibility of the underlying choroidal vasculature; (2) SD-OCT findings of increased signal transmission in the choroid in the absence of RPE; or (3) reduced autofluorescence signal on autofluorescence images.

### Statistical analysis

A chi-square test was performed for comparing the categorical variables between the groups and student’s t-test for comparing the continuous variables. Stepwise multivariate logistic regression analysis was performed to identify the relationship between neovascular AMD without exudative recurrence during the maintenance phase and the patients’ baseline clinical characteristics. SPSS software, version 18.0 (SPSS Inc., Chicago, IL, USA), was used for all statistical analyses; *P*-values < 0.05 were considered statistically significant.

## Results

In total, 608 eyes with neovascular AMD that had been followed up for 24 months were initially identified from the database. Among these, 216 eyes were excluded due to treatment with the TAE regimen (*n* = 178), foveal scar or atrophy at baseline (*n* = 32), and other concurrent macular diseases including diabetic retinopathy or retinal vein occlusion (*n* = 6).

The mean age of the study group was 69.8 ± 8.4 years, and the average number of anti-VEGF injections administered in all the patients during the 24-month study period was 9.3 ± 4.8 (range, 3–17). All the patients were South Korean. Table 1 presents detailed clinical data of the enrolled patients.

**Table 1:** Baseline characteristics of patients.

	Total eyes (*n* = 392)	Exudative recurrence during a 2-year anti-VEGF treatment		*P*
		Yes (n = 334)	No (n = 58)	
**Age (years)**, mean ± SD	69.8 ± 8.4	70.0 ± 9.1	63.6 ± 8.8	0.013^*a*^
**Gender**, *n* (%)				0.160^*b*^
Male	210 (53.6%)	174 (52.1%)	36 (62.1%)	
Female	182 (46.4%)	160 (47.9%)	22 (37.9%)	
**Mean baseline BCVA (logMAR)** (Snellen equivalent)	0.51 ± 0.45 (20/64)	0.52 ± 0.46 (20/66)	0.48 ± 0.44 (20/60)	0.071^*a*^
**Central foveal thickness (µm)**, mean ± SD	432 ± 214	435 ± 228	409 ± 201	0.336^*a*^
**Subfoveal choroidal thickness (µm)**, mean ± SD	279 ± 109	281 ± 133	277 ± 115	0.681^*a*^
**Lesion location**, *n* (%)				0.316^*b*^
Subfoveal	260 (66.3%)	223 (66.7%)	37 (63.8%)	
Juxtafoveal	81 (20.7%)	71 (21.3%)	10 (17.2%)	
Extrafoveal	51 (13.0%)	40 (12.0%)	11 (19.0%)	
**Lesion size (mm**^**2**^**)**, mean ± SD	2.19 ± 1.82	2.25 ± 1.88	1.08 ± 0.81	0.008^*a*^
**Choroidal vascular hyperpermeability**, n (%)^*c*^	144 (36.7%)	118 (35.3%)	26 (44.8%)	0.166^*b*^
**Baseline fluid feature**, *n* (%)				0.073^*b*^
SRF alone	121 (30.9%)	97 (29.0%)	24 (41.4%)	
SRF with other exudation (IRF, hemorrhage, or SHRM)	137 (34.9%)	116 (34.7%)	21 (36.2%)	
Absence of SRF	134 (34.2%)	121 (36.3%)	13 (22.4%)	
**PED at baseline**, *n* (%)				
Fibrovascular PED	267 (68.1%)	235 (70.4%)	32 (55.2%)	0.022^*b*^
Serous PED	91 (23.2%)	75 (22.4%)	16 (27.5%)	0.085^*b*^
**PED height at baseline (µm)**, mean ± SD	269 ± 193	274 ± 186	244 ± 205	0.326^*a*^
**MNV subtype**, *n* (%)				0.088^*b*^
Type 1	163 (41.5%)	146 (43.7%)	17 (29.3%)	
Aneurysmal type 1/PCV	134 (34.2%)	106 (31.7%)	28 (48.3%)	
Type 2	46 (11.8%)	40 (12.0%)	6 (10.3%)	
Type 3	49 (12.5%)	42 (12.6%)	7 (12.1%)	
**Anti-VEGF agent**, *n* (%)				< 0.001^*b*^
Ranibizumab	85 (21.6%)	65 (19.6%)	20 (34.5%)	
Aflibercept	225 (57.4%)	187 (56.0%)	38 (65.5%)	
Both^*d*^	82 (20.9%)	82 (24.4%)	0 (0%)^*e*^	
**Number of anti-VEGF injections**, mean ± SD	9.3 ± 4.8	10.2 ± 5.3	3.0 ± 0.0^*f*^	< 0.001^*a*^

*AMD* age-related macular degeneration, *BCVA* best-corrected visual acuity, *IRF* intraretinal fluid, *logMAR* logarithm of the minimum angle of resolution, *MNV* macular neovascularization, *PCV* polypoidal choroidal vasculopathy, *PED* pigment epithelial detachment, *SD* standard deviation, *SHRM* subretinal hyperreflective material, *SRF* subretinal fluid, *VEGF* vascular endothelial growth factor.

^*a*^Student’s t-test.

^*b*^Chi-square test.

^*c*^Of all eyes, 39 indocyanine angiographic images were not available.

^*d*^Patients who switched from one anti-VEGF drug to another during the study period.

^*e*^No patients switched anti-VEGF after the first remission in the group without recurrence because they were not administered additional anti-VEGF after the three loading injections.

^*f*^All patients without recurrence received only three loading injections during the 24-month follow-up.

### Neovascular AMD without exudative recurrence during the maintenance phase

Among the 392 eyes included, 329 (83.9%) showed initial remission after the three loading injections. Moreover, 58 eyes (14.8%) showed no exudative recurrence after the initial loading injection during the 24-month follow-up and did not need additional injections. Exudative recurrence was observed in 334 eyes (85.2%) more than once during the 24-month maintenance phase.

Several significant differences in the baseline characteristics were observed between the groups with (*n* = 334) and without (*n* = 58) exudative recurrence. Patients without recurrence were significantly younger in age than those with recurrence (63.6 ± 8.8 years vs. 70.0 ± 9.1 years; *P* = 0.013) (Table 1). The mean lesion size of the eyes without exudative recurrence was significantly smaller than that of the eyes with exudative recurrence (1.08 ± 0.81 mm^2^ vs. 2.25 ± 1.88 mm^2^; *P* = 0.008) (Table 1). Fibrovascular PED was less frequent in eyes without exudative recurrence than in those with exudative recurrence (55.2% vs. 70.4%; *P* = 0.022) (Table 1). The mean number of injections administered was significantly lower in the group without recurrence than in the group with exudative recurrence, as the former did not receive additional injections after the three loading injections during the 24-month period (3.0 ± 0.0 vs. 10.2 ± 5.3; *P* < 0.001) (Table 1).

There were no differences in sex, mean baseline BCVA, mean central subfoveal thickness, mean subfoveal choroidal thickness, lesion location, presence of choroidal vascular hyperpermeability, baseline fluid features, or MNV subtype between the two groups (Table 1).

### Visual outcomes

**Figure 1:**
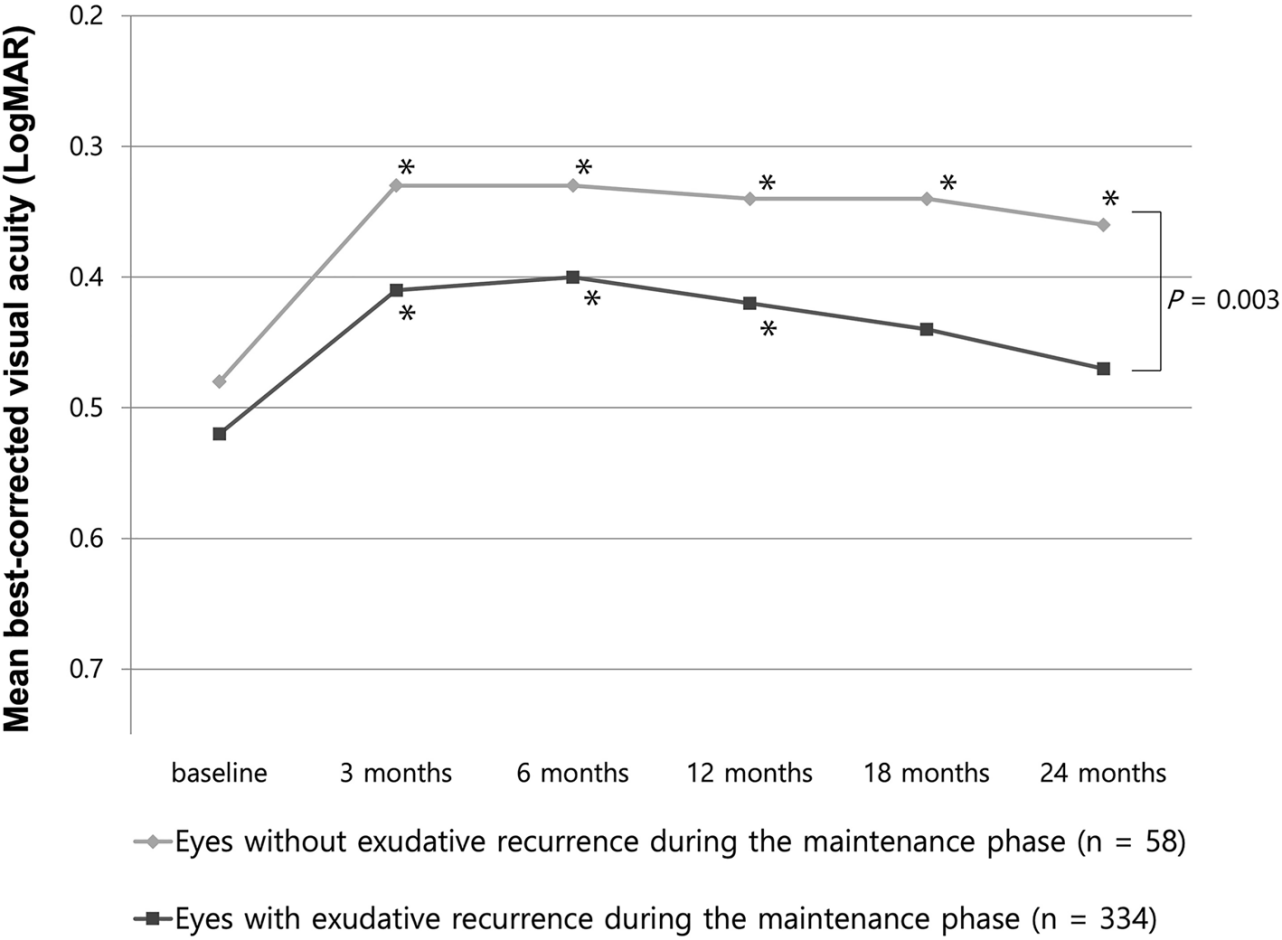
Mean best-corrected visual acuity (BCVA) of the groups with and without exudative recurrence during the 24-month follow-up. The improved visual acuity after three loading injections was maintained in the eyes without exudative recurrence during the 24 months, whereas that of the eyes with exudative recurrence showed a gradual decline (*P < 0.05, compared with baseline BCVA). At 24 months, the eyes without exudative recurrence showed significantly improved BCVA than those having exudative recurrence more than once (*P* = 0.003).

The time course of the BCVA changes was compared between the eyes with and without exudative recurrence during the 24-month follow-up (Fig. 1). The visual gain after the three loading injections tended to be maintained in the eyes without exudative recurrence for 24 months, whereas that of the eyes with exudative recurrence showed a gradual decline, and eventually, the BCVA showed no significant difference compared to that at baseline (Fig. 1). After the 24-month follow-up, the BCVA of the eyes without recurrence was significantly better than that of the eyes with recurrence during the maintenance phase (0.35 ± 0.30 [Snellen equivalent 20/44] vs. 0.47 ± 0.29 [20/59]; *P* = 0.003) (Fig. 1).

No significant difference was found in the proportion of improved visual acuity (gain of three or more lines in BCVA) between the eyes with and without exudative recurrence at 24 months. On the contrary, the proportion of eyes showing worsening of more than three lines of visual acuity in the group without exudative recurrence was significantly lower than that in the group with exudative recurrence (5.2% [3/58 eyes] vs. 15.3% [51/334 eyes]; *P* = 0.039) (Table 2). The proportion of eyes with 20/40 or better vision at 24 months tended to be higher in the group without recurrence than in the group with recurrence, without a significant difference between them (44.8% vs. 33.2%; *P* = 0.083) (Table 2).

**Table 2:** Treatment outcomes after the 2-year anti-VEGF treatment.

	Total eyes (*n* = 392)	Eyes with exudative recurrence during the maintenance phase (334 eyes)	Eyes without exudative recurrence during the maintenance phase (58 eyes)	*P*
**BCVA at 24 months (logMAR [Snellen equivalent])**, mean ± SD	0.44 ± 0.31 (20/55)	0.47 ± 0.29 (20/59)	0.35 ± 0.30 (20/44)	0.003^*a*^
**Central foveal thickness at 24 months (µm)**, mean ± SD	292 ± 154	297 ± 186	268 ± 133	0.035^*a*^
**BCVA ≥ 20/40**, *n* (%)	137 (34.9%)	111 (33.2%)	26 (44.8%)	0.087^*b*^
**BCVA ≤ 20/200**, *n* (%)	44 (11.2%)	40 (12.0%)	4 (6.9%)	0.258^*b*^
**BCVA changes**, *n* (%)				
Improved ≥ 3 lines (logMAR 0.3)	130 (33.2%)	118 (35.3%)	16 (27.6%)	0.251^*b*^
Worsened ≥ 3 lines (logMAR 0.3)	54 (13.8%)	51(15.3%)	3 (5.2%)	0.039^*b*^
**Development of macular atrophy**, *n* (%)	78 (19.9%)	73 (21.9%)	5 (8.6%)	0.020^*b*^

*BCVA* best-corrected visual acuity, *logMAR* logarithm of the minimum angle of resolution, *SD* standard deviation, *VEGF* vascular endothelial growth factor.

^*a*^Student’s t-test.

^*b*^Chi-square test.

### Anatomical outcomes

**Figure 2:**
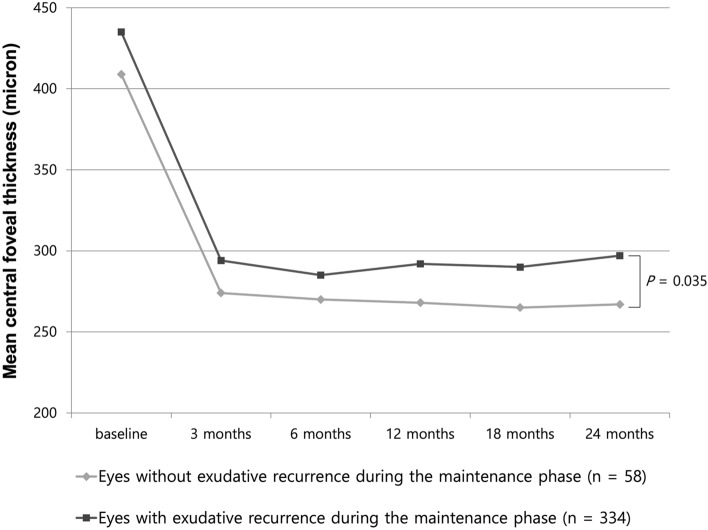
Central foveal thickness of the groups with and without exudative recurrence during the 24-month follow-up. The central foveal thickness of both the groups was maintained after the initial three loading injections during the 24 months. The central foveal thickness of the eyes without exudative recurrence was significantly lesser than that of the eyes with recurrence after 24 months (*P* = 0.035).

The mean central foveal thickness significantly decreased during the 24-month follow-up in both groups (Fig. 2). After 24 months of treatment, the mean central foveal thickness of the eyes without exudative recurrence was significantly lesser than that of the eyes with exudative recurrence during the maintenance phase (268 ± 133 µm vs. 297 ± 186 µm; *P* = 0.035) (Fig. 2). The incidence of MA development was significantly lower in the eyes without exudative recurrence than in the eyes with exudative recurrence (8.6% [5/58 eyes] vs. 21.9% [73/334 eyes]; *P* = 0.020) (Table 2).

### Odds for no exudative recurrence during the maintenance phase

Several significant baseline characteristics associated with the absence of exudative recurrence were identified using the multivariate logistic regression analysis (Table 3). Younger age at baseline (odds ratio [OR], 0.901; 95% confidence interval [CI], 0.831–0.979; *P* = 0.033) was associated with increased odds of no recurrence during the maintenance phase. Similarly, smaller lesion size (OR, 0.589; 95% CI, 0.383–0.901; *P* = 0.016) and absence of fibrovascular PED (OR, 1.349; 95% CI, 1.226–2.884; *P* = 0.028) were significantly associated with an increased likelihood of the absence of recurrence. However, serous PED was not significantly associated with absence of recurrence in the maintenance phase (*P* = 0.652). Other factors, including sex, baseline BCVA, baseline subfoveal choroidal thickness, presence of choroidal vascular hyperpermeability, baseline fluid features, PED height, MNV subtype, and type of anti-VEGF, showed no correlation with the absence of recurrence (Table 3).

**Table 3:** Association between baseline characteristics and the absence of exudative recurrence: logistic regression analysis.

Variable	Univariate analysis			Multivariate analysis	
	OR (95% CI)	*P*	OR (95% CI)		*P*
**Age**	0.833 (0.811–0.985)	0.021	0.901 (0.831–0.979)		0.033
**Gender**^*a*^	0.981 (0.875–1.037)	0.721			
**Baseline BCVA (logMAR)**	1.353 (0.737–2.142)	0.329			
**Baseline central foveal thickness**	0.917 (0.865–1.118)	0.337			
**Baseline subfoveal choroidal thickness**	1.009 (0.821–1.623)	0.312			
**Lesion location**^*a*^	0.891 (0.721–2.315)	0.343			
**Lesion size**	0.557 (0.361–0.859)	0.008	0.589 (0.383–0.901)		0.016
**Presence of choroidal vascular hyperpermeability**^*a*^	1.442 (0.815–2.111)	0.656			
**Baseline fluid feature**^*a*^		0.317			
SRF alone	1.251 (0.876–1.943)	0.055			
SRF with IRF, retinal hemorrhage, and/or SHRM	0.658 (0.325–1.734)	0.246			
Absence of SRF	1.00				
**Presence of PED**^*a*^		0.024			0.038
None	1.571 (1.135–3.165)	0.019	1.349 (1.226–2.884)		0.028
Fibrovascular PED	1.00		1.00		
Serous PED	0.834 (0.307–2.265)	0.721	0.914 (0.416–2.881)		0.652
**PED height (µm)**^*a*^		0.054			
< 100	1.325 (1.052–2.921)	0.008			
100–200	1.136 (0.973–1.856)	0.343			
> 200	1.00				
**MNV subtype**^*a*^		0.140			
Type 1	1.00				
Aneurysmal type 1/PCV	1.472 (0.698–3.378)	0.408			
Type 2	1.784 (0.642–3.992)	0.267			
Type 3	1.821 (0.991–4.121)	0.082			
**Anti-VEGF agent (ranibizumab or aflibercept)**^*a*^	0.897 (0.771–1.313)	0.469			

*AMD* age-related macular degeneration, *BCVA* best-corrected visual acuity, *CI* confidence interval, *logMAR* logarithm of the minimum angle of resolution, *MNV* macular neovascularization, *OR* odds ratio, *PCV* polypoidal choroidal vasculopathy, *PED* pigment epithelial detachment, *SRF* subretinal fluid, *VEGF* vascular endothelial growth factor.

^*a*^Categorical variable.

## Discussion

In this study, only patients treated with the PRN regimen were included because a proactive treatment such as the TAE regimen could decrease the recurrence rate of neovascular AMD^20^. Hence, our study identified and analyzed the characteristics of a stable subset of neovascular AMD cases that did not show recurrence after initial remission during the long-term follow-up.

Approximately 15% of the patients with neovascular AMD did not require additional anti-VEGF injections after the initial three loading injections during the 24-month follow-up. Despite administering only three anti-VEGF injections in 2 years, patients without exudative recurrence demonstrated better visual and anatomical outcomes and a lower incidence of MA than those with exudative recurrence.

Similar to our results, several previous studies reported stable neovascular AMD cases that did not show recurrence after the initial loading injections. In the SUSTAIN study, which applied the PRN regimen in 513 patients, approximately 20% of the patients did not require an injection after the first three treatments during the 12-month follow-up^21^. In another retrospective study of 139 eyes, 25.2% of the patients presenting dry macula after the loading treatment did not need additional injections during the 24-month follow-up^22^. In the EVEREST study, 14.1% of PCV patients in the ranibizumab monotherapy group did not require additional anti-VEGF injections after the three loading injections during 12 months^23^.

In neovascular AMD patients who do not experience recurrence after the initial loading injection, the TAE or fixed regimen might involve unnecessary anti-VEGF injections and increased treatment burden. Furthermore, although debatable, there are concerns that an increased number of anti-VEGF injections may lead to MA progression^9^. Therefore, the prediction that stable neovascular AMD shows infrequent recurrence could assist the clinicians in managing patients in an individualized manner. In this study, several baseline characteristics relevant to stable neovascular AMD without recurrence during the 24-month follow-up were identified.

Infrequent exudative recurrence correlated with younger age and smaller lesion size. A previous study reported that younger age was associated with infrequent recurrence of neovascular AMD^22^. Additionally, the CNV size correlates with visual prognosis. There is evidence regarding an association between smaller CNV lesions and better visual acuity outcomes in several phase 3 trials^24,25^. Furthermore, smaller CNV areas were associated with a decreased risk of exudative recurrence, particularly IRF development, during anti-VEGF treatment^26^. In addition to these lines of evidence regarding the favorable prognosis of smaller CNV lesions, our results suggest that during anti-VEGF treatment, consideration of lesion size could be a predictor for recurrence and can assist in deciding the treatment regimen after the loading injections.

The absence of fibrovascular PED was identified as another predictive factor for the low recurrence risk during the long-term follow-up. On the other hand, the presence of serous PED was not significantly associated with recurrence. It has been reported that PED is associated with an increased treatment frequency^27^. Particularly, PED at baseline was more likely to cause recurrence and activity when shifting from a fixed dosing regimen to a flexible dosing regimen^12^. More recently, it was reported that a higher PED height increases the risk of IRF development during anti-VEGF treatment^26^. Hence, neovascular AMD with fibrovascular PED should be monitored cautiously. Moreover, the PRN regimen might be insufficient to preserve the visual gain obtained after the first remission.

The frequency of recurrence during anti-VEGF treatment may be affected by the subtype of neovascular AMD, because the response to anti-VEGF varies slightly among the neovascular AMD subtypes^28^. Previously, several studies reported that the subtype of neovascular AMD is associated with the frequency of exudative recurrence. Kuroda et al. reported that PCV tended to recur frequently after the loading injection^22^. Compared to the other types of MNV, RAP (type 3 MNV) could reportedly achieve a long remission period of 6 or 12 months without recurrence^29,30^. However, in our study, the subtype of neovascular AMD did not demonstrate any association with the absence of recurrence after initial remission. This could be attributed to the difference between the remission periods of each study; our study evaluated a longer remission period of 24 months compared to that of previous studies^29,30^. Since type 3 MNV is almost reactivated within 7–12 months after the loading injection^28^, it did not show a difference in the recurrence frequency as compared to the other MNV types during the 24-month follow-up.

Our study has several limitations, including its retrospective design. A large proportion of participants treated with the TAE regimen were excluded, which possibly introduced a bias. Future studies should evaluate the association between the frequency of exudative recurrence and TAE regimen. Furthermore, studies should be performed to determine the optimal strategy to manage patients with neovascular AMD, who are anticipated to experience infrequent recurrence after initial remission. Additionally, the stability of neovascular AMD, which does not recur, should be evaluated over a longer follow-up period.

In conclusion, 14.8% of the eyes with neovascular AMD in our study showed no exudative recurrence during the 24-month follow-up and did not require additional anti-VEGF injections after the initial three loading injections. Younger age, smaller lesion size, and absence of fibrovascular PED correlated with infrequent recurrence after initial remission. Identifying the predictive factors at baseline might be clinically relevant, as this information allows clinicians to categorize patients better and provide individualized anti-VEGF treatments.

## Data Availability

The datasets used and analyzed in this study will be available from the corresponding author on reasonable request.
